# Supplementary figures and images for: Space-filling and benthic competition on coral reefs
Source: PeerJ. 2021 Jun 29;9:e11213. doi: 10.7717/peerj.11213 (PMC8253116; doi:10.7717/peerj.11213)

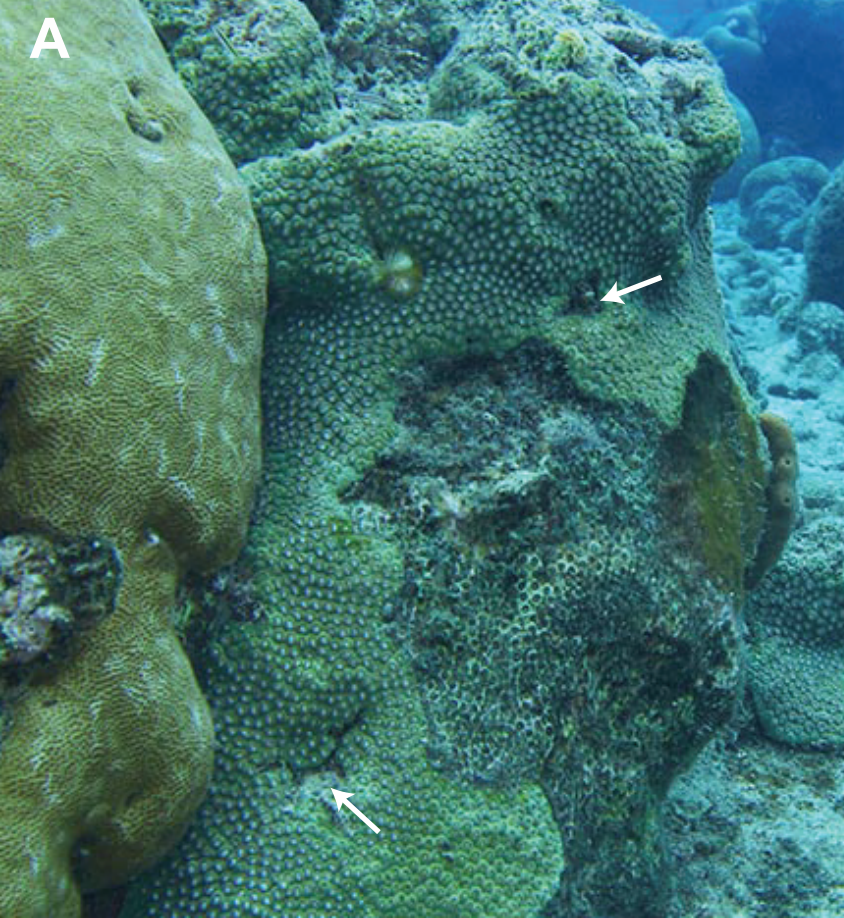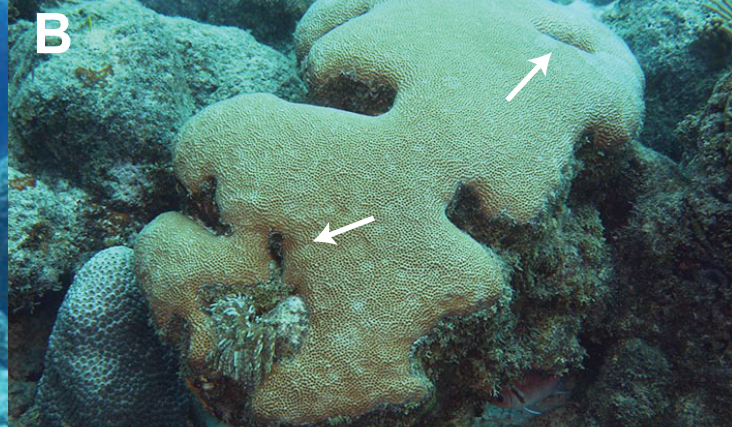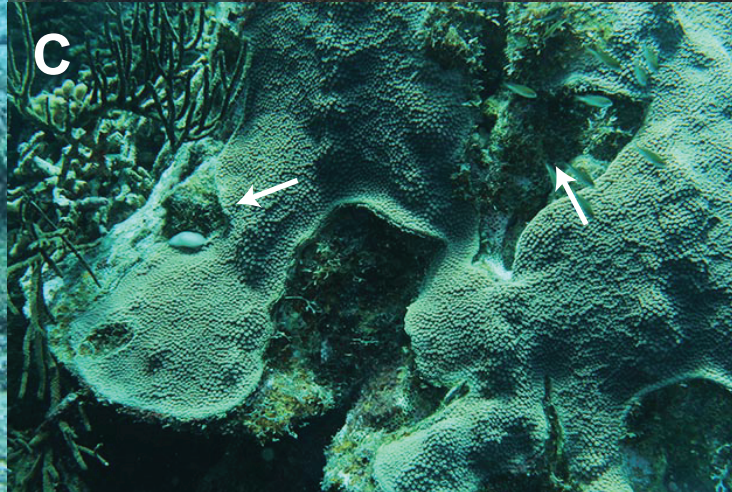

Supplement: Supplemental Information 3 — (A) Montastraea cavernosa. (B) Siderastrea siderea. (C) Orbicella faveolata. Patches are indicated with white arrows. [file peerj-09-11213-s003.pdf]

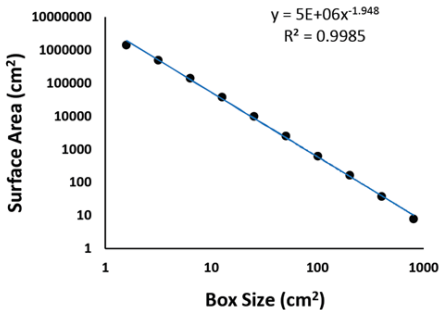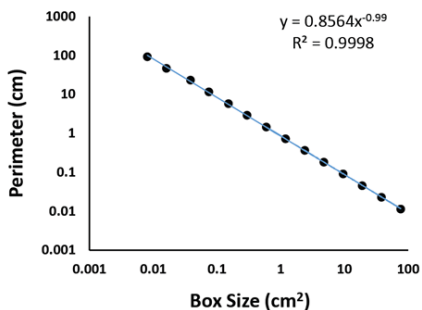

Supplement: Supplemental Information 4 — Surface area (left) and perimeter length (right) of an Orbicella faveolata colony over multiple scales using a box-counting algorithm. A linear regression was applied to the logged values of the variables. The parameters are displayed for the associated power function model for the original values of the variables. The space-filling dimension is equal to the exponent of the power function changed to a positive value, Eq. S1. [file peerj-09-11213-s004.pdf]

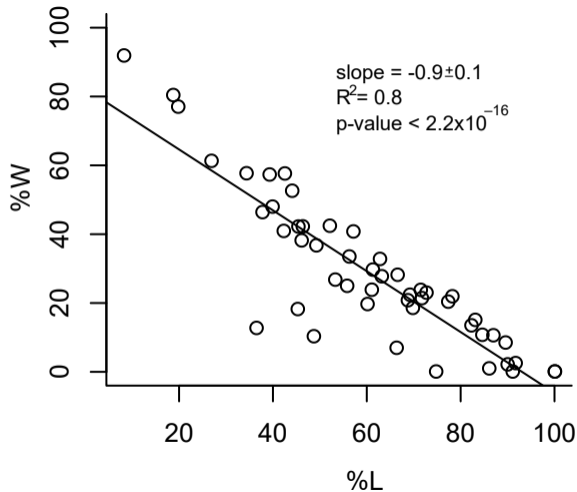

Supplement: Supplemental Information 5 — The percentages of winning perimeters (%W) plotted against the percentage of losing perimeter (%L) for each coral colony. The solid line corresponds to the linear best-fit using least-squares method. The legend provides the slope, R-squared value, and p-value. [file peerj-09-11213-s005.pdf]

**A**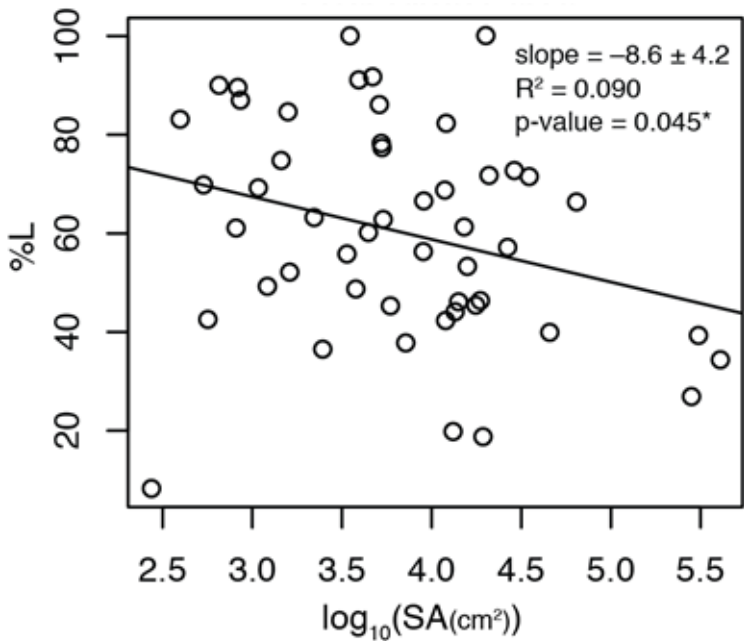**B**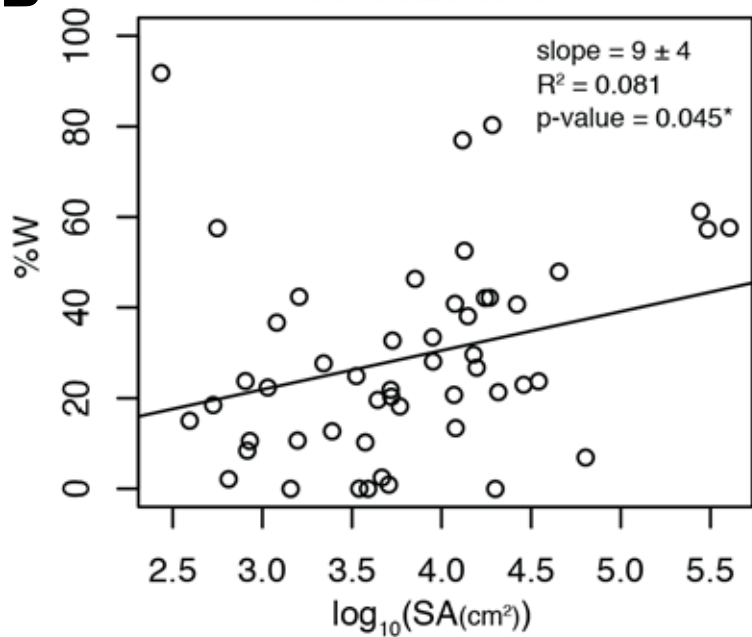

Supplement: Supplemental Information 6 — (A) Percentage of losing outcomes (%L). (B) Percentage of wining outcomes (%W). The solid line corresponds to the linear best-fit using least-squares method. The legend provides the slope, R-squared value, and p-value. [file peerj-09-11213-s006.pdf]

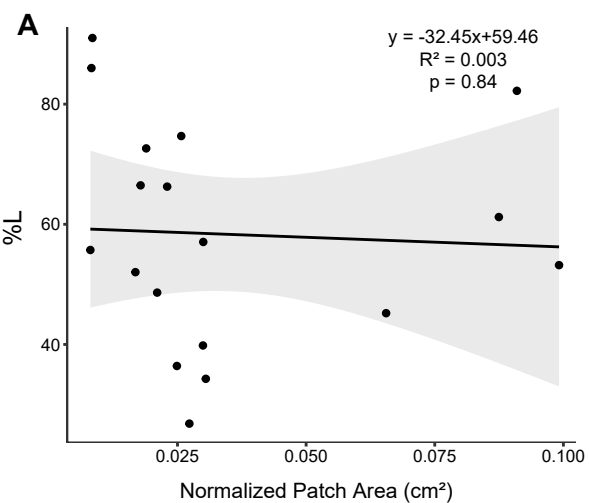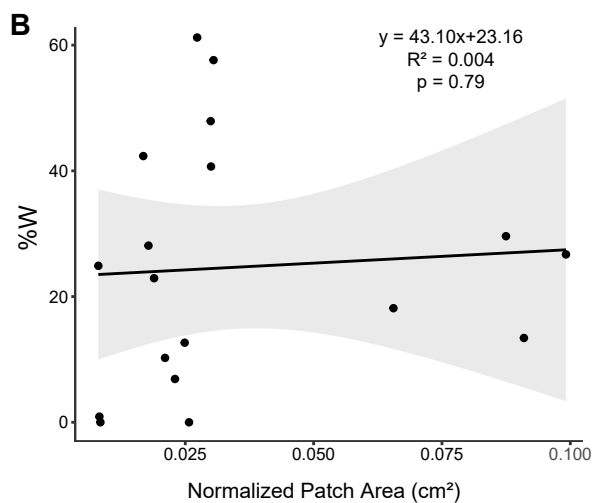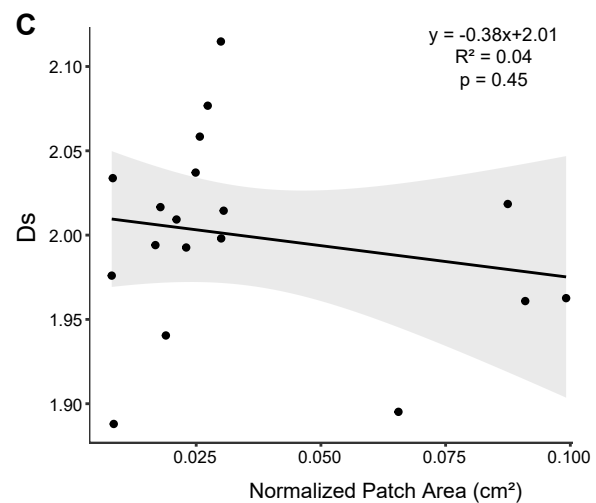

Supplement: Supplemental Information 8 — (A) Percentage of losing perimeter (%L) vs normalized patch area (dead tissue area divided by coral surface area). (B) Percentage of winning perimeter (%W) vs normalized patch area. (C) Space-filling dimension (Ds) vs normalized patch area. [file peerj-09-11213-s008.pdf]

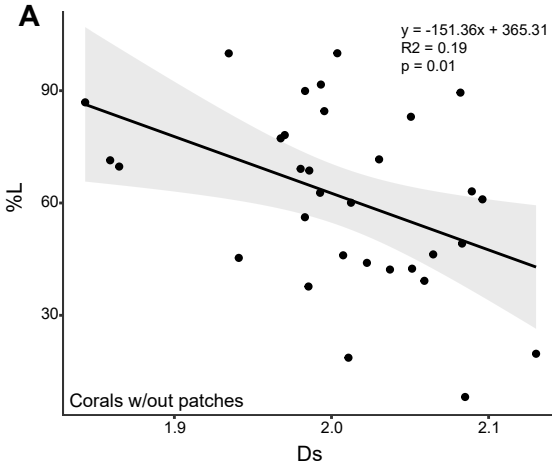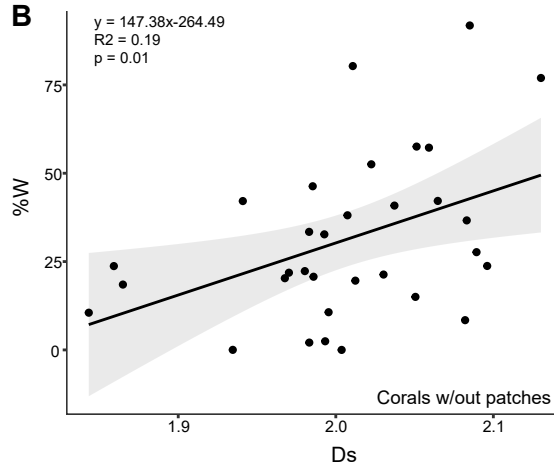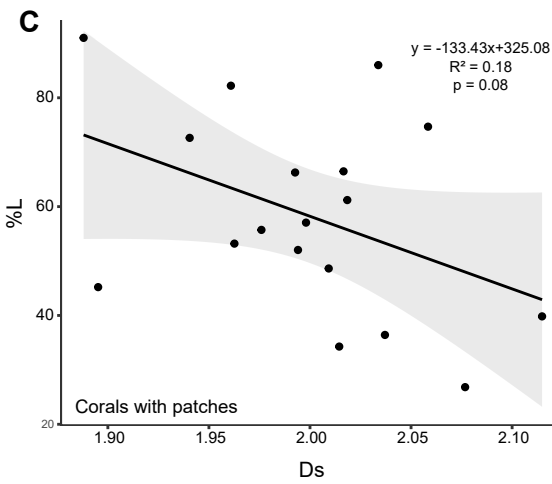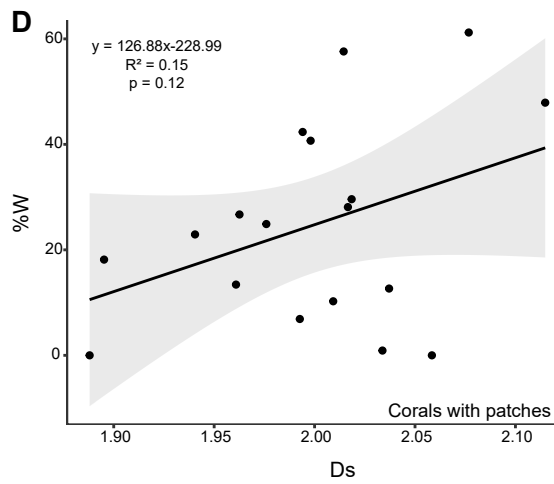

Supplement: Supplemental Information 9 — (A) Percentage of losing perimeter (%L) vs space-filling dimension of coral without patches. (B) Percentage of winning perimeter (%W) vs space-filling dimension of coral without patches. (C) Percentage of losing perimeter (%L) vs space-filling dimension of coral with patches. (D) Percentage of winning perimeter (%W) vs space-filling dimension of coral with patches. [file peerj-09-11213-s009.pdf]
