# Supplementary material for: Space-filling and benthic competition on coral reefs
Source: PeerJ. 2021 Jun 29;9:e11213. doi: 10.7717/peerj.11213 (PMC8253116; doi:10.7717/peerj.11213)
Supplement: Supplemental Information 7 — (A) Orbicella faveolata. (B) Montastraea cavernosa. (C) Siderastrea siderea. (D) Pseudodiploria strigosa. The solid line corresponds to the linear best-fit using least-squares method. The legend provides the slope, R-squared value, and p-value. [file peerj-09-11213-s007.pdf]

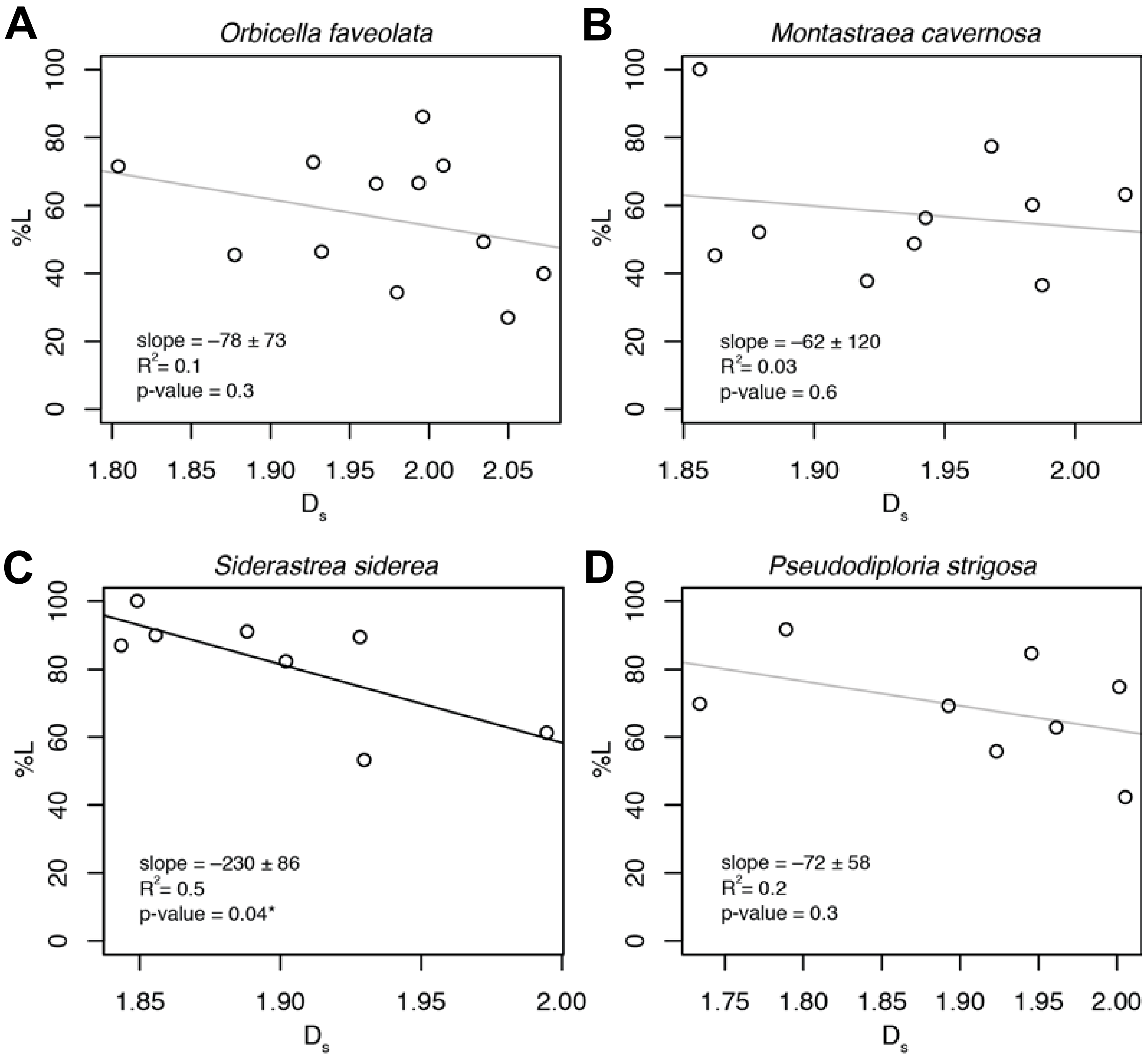
A
Orbicella faveolata
%L
D_s
slope = −78 ± 73
R² = 0.1
p-value = 0.3
B
Montastraea cavernosa
slope = −62 ± 120
R² = 0.03
p-value = 0.6
C
Siderastrea siderea
slope = −230 ± 86
R² = 0.5
p-value = 0.04*
D
Pseudodiploria strigosa
slope = −72 ± 58
R² = 0.2
p-value = 0.3
