# Supplementary material for: Space-filling and benthic competition on coral reefs
Source: PeerJ. 2021 Jun 29;9:e11213. doi: 10.7717/peerj.11213 (PMC8253116; doi:10.7717/peerj.11213)
Supplement: Supplemental Information 10 [file peerj-09-11213-s010.docx]

**Table S1.** Validation of 2D and 3D Box Count algorithms

| Shape | Dimension | Measurement | Error |
| --- | --- | --- | --- |
| Circle | 1.000 | 1.009 | 0.90% |
| Koch Curve | 1.262 | 1.279 | 1.36% |
| Sierpinski Triangle | 1.585 | 1.565 | -1.26% |
| Kidney Vasculature | 1.610 | 1.627 | 1.06% |
| Menger Sponge | 2.727 | 2.822 | 3.49% |
