# Supplementary material for: Space-filling and benthic competition on coral reefs
Source: PeerJ. 2021 Jun 29;9:e11213. doi: 10.7717/peerj.11213 (PMC8253116; doi:10.7717/peerj.11213)
Supplement: Supplemental Information 11 [file peerj-09-11213-s011.docx]

**Table S2. Statistical analysis for percentage perimeter losing (%L), neutral (%N), and winning (%W)**

| Statistics | %L | %N | %W |
| --- | --- | --- | --- |
| Minimum | 8.183 | 0.000 | 0.00 |
| 1^st^ quartile | 45.238 | 3.486 | 12.85 |
| Median | 61.092 | 8.342 | 23.74 |
| Mean | 60.464 | 10.699 | 28.84 |
| 3^rd^ quartile | 76.624 | 13.911 | 41.81 |
| Maximum | 100.00 | 50.921 | 91.82 |
