# Supplementary material for: Space-filling and benthic competition on coral reefs
Source: PeerJ. 2021 Jun 29;9:e11213. doi: 10.7717/peerj.11213 (PMC8253116; doi:10.7717/peerj.11213)
Supplement: Supplemental Information 12 [file peerj-09-11213-s012.doc]

**Supplemental Methods**

*Field sampling*

The perimeters and surface areas of 50 coral colonies were imaged in reef sites around the Caribbean island of Curaçao: East Point, CARMABI, Water Factory, Snake Bay, and Westpunt. Sampling was conducted in April of 2015, November of 2015, and April of 2016.

*2D perimeter models and competition outcomes*

Each colony was photographed at a close distance (~10 cm) with 40–50% overlap between neighboring images to obtain a high resolution of the perimeter (Figure 1C). For the 3D coral reconstructions, each coral colony was photographed from various angles and distances (30 cm – 1 m), obtaining, per colony, from 40 images (small colonies) to 150 images (large colonies). All images were stored in 72 dpi JPEG format. The length/pixel resolution depended on the distance with respect to the coral. The scale was set using an in-reef ruler visible in the imagery. The ruler was placed along the interface of the coral colony and included in at least four whole colony images. The series of overlapping perimeter images were started at the ruler and continued around the perimeter until the ruler was reached. This allowed all perimeter images to be scaled by one ruler image. The density of pixels ranged from 5.7 to 57.6 pixels/mm, leading to a minimum threshold resolution of ~0.5 mm. This allowed a multi-scale analysis of the perimeter length.

The digital perimeters were imported in MATLAB (MATLAB R2015b, The MathWorks Inc., Natick, MA, 2000), and the percentage of losing (%Losing), winning (%Winning), and neutral (%Neutral) interactions in a perimeter were calculated, respectively, as the percentage of red pixels (losing), green pixels (winning), and blue pixels (neutral) in the RGB channels.

*3D coral models*

The photographs from 50 coral colonies were processed using the photogrammetry software Autodesk® ReMake®, 2016. This approach was previously tested in Naumann et al., 2009 and Lavy et al., 2015 (Naumann et al., 2009; Lavy et al., 2015). In this software, point clouds generated from imagery collected in the field were overlaid with texturized meshes to create 3D coral models (Burns et al., 2015; Leon et al., 2015). The 3D models scale was calibrated using in-reef PVC rulers and defects in the meshes were manually filled (holes) or removed (artifacts). Mesh regions that did not belong to corals were removed from the final 3D object so that only coral mesh remained (Figures 3 and S1).

*Perimeter and surface space-filling dimensions*

The box-counting dimension, a proxy for space-filling dimension, was calculated using a box counting method (Falconer, 2003). The algorithm was generalized to n-dimensions and applied to 2D and 3D models. The initial box occupied the whole n-volume and had the same aspect ratio as the input data. The size of the box, G, was bisected at every step of the algorithm. For each step, N is the number of boxes containing points of the object. This number N decreases with the box size G following the power law N = MG^–D^. Here D is the box-counting dimension and M is the geometric scale prefactor (Falconer, 2003). The logarithmic transformation of this relationship leads to the linear equation

|  | . | (S1) |
| --- | --- | --- |

At each step, boxes were bisected to generate the new set of boxes. This linear model was fitted using the least squares method; the box-counting dimension *D* was extracted from the slope as illustrated in Figure S2. The 95% confidence intervals for the box-counting dimension (slope of the log-log transformed data) was calculated using a Monte-Carlo non-parameteric bootstrap resampling method; the set of points used was close to but less than the full bootstrap set size (n^n, where n is the number of bisections), and it was commensurate with the optimal processor bundling size for the parallelized CUDA code (Efron & Tibshirani, 1994). The code is available on GitHub: https://github.com/luquelab/George_Mullinix_etal_2021.

The box-counting analysis for the perimeter was obtained from the 2D high-resolution images of the projected coral boundaries. The original RGB files were converted to binary format in MATLAB, where pixels forming the perimeter received a value of 1, while the rest of the pixels received a value of 0. The box counting method described above was applied, Eq. (S1). The resolution of the perimeters allowed a minimum of 10 iterations, resulting in a multi-scale analysis of three orders of magnitude (2^10^ = 1024).

The surface box-counting dimension was calculated from the 3D digital coral models. The cloud mesh was tessellated using triangles, and the position of the vertices and orientation of each triangle were stored in a STereoLithograpy (STL) file using Autodesk® Remake®, 2016. The STL files were read in by C-code. The mesh was extracted as a list of points. The box-counting dimensions were calculated using the box counting method described above, Eq. (S1). The resolution of the 3D digital models allowed a minimum of five bisections (iterations) in the box counting algorithm and the total scaling factor was ≥ 2^5^ = 32.

*Coral geometric properties: perimeter, surface area, volume, and polyp size*

The 3D digital coral models were used to measure the perimeter, surface area, and volume for each colony. The perimeter, surface area, and volume were calculated with the mesh report tool in Autodesk® Remake®, 2016. The 3D photogrammetry software was shown to have <10% volumetric error and <20% surface area error in Lavy et al., 2015.

The 2D digital coral models were used to measure the perimeter at a higher resolution (0.05 mm to 0.5 mm). A 1 mm ruler-resolution was applied using the Richardson divider algorithm. The length of the perimeter, *P*, was calculated by ‘walking’ a ruler of length, *R*, and summing up all the steps. The perimeter was obtained as an average over 10 iterations of the algorithm starting from different points along the perimeter.

Supporting Data Set 1: Excel spreadsheet containing the biological and geometrical properties of each coral colony. The columns correspond to 3D model name, ID, coral species, morphology type, depth (feet), polyp diameter (cm), volume (cm^3^), surface area (cm^2^), volume-to-surface area ratio (Volume_SA), surface-area-to-polyp area ratio (SA_polyp2), perimeter space-filling dimension (Perimeter_D), perimeter space-filling dimension at lower confidence interval (Perimeter_D Lower CI), perimeter space-filling dimension at upper confidence interval (Perimeter_D Upper CI), surface space-filling dimension (Surface_D), surface space-filling dimension at lower confidence interval (Surface_D Lower CI), surface space-filling dimension at upper confidence interval (Surface_D Upper CI), 2D perimeter length obtained from Richardson's algorithm (RP_length; cm), perimeter length obtained from 3D models (perimeter_length_3D; cm), 3D perimeter-to-polyp size ratio (P_polyp_3D); 2D perimeter-to-surface area ratio (RP_SA), 3D perimeter-to-surface area ratio (P_SA_3D), percentage of losing perimeter (L), percentage of winning perimeter (W), percentage of neutral perimeter (N), region of Curacao where site was located (Area), colony dead tissue area (Dead_tissue_area; cm^2^), dead tissue area-to-surface area (Normalized_dead_tissue_area).

Supporting Data Set 2: Excel spreadsheet containing the univariate analysis results including Pearson Correlations between the percentages of losing (%L) or winning (%W) perimeter and the biological/geometrical variables.**References**

Burns JHR, Delparte D, Gates RD, Takabayashi M. 2015. Integrating structure-from-motion photogrammetry with geospatial software as a novel technique for quantifying 3D ecological characteristics of coral reefs. *PeerJ* 2015:e1077.

Efron B, Tibshirani R. 1994. An introduction to the bootstrap. Boca Raton: CRC Press.

Falconer KJ. 2003. *Fractal geometry : mathematical foundations and applications*. Hoboken: John Wiley & Sons.

Lavy A, Eyal G, Neal B, Keren R, Loya Y, Ilan M. 2015. A quick, easy and non-intrusive method for underwater volume and surface area evaluation of benthic organisms by 3D computer modelling. *Methods in Ecology and Evolution* **6**:521-31.

Leon JX, Roelfsema CM, Saunders MI, Phinn SR. 2015. Measuring coral reef terrain roughness using ‘Structure-from-Motion’ close-range photogrammetry. *Geomorphology* 242:21–28.

Naumann MS, Niggl W, Laforsch C, Glaser C., Wild C. 2009. Coral surface area quantification–evaluation of established techniques by comparison with computer tomography. *Coral Reefs* 28:109–117.
